# Effects of Labelling and Increasing the Proportion of Lower-Energy Density Products on Online Food Shopping: A Randomised Control Trial in High- and Low-Socioeconomic Position Participants

**DOI:** 10.3390/nu12123618

**Published:** 2020-11-25

**Authors:** Lucile Marty, Brian Cook, Carmen Piernas, Susan A. Jebb, Eric Robinson

**Affiliations:** 1 Department of Psychological Sciences, University of Liverpool, Liverpool L69 7ZA, UK; 2 Nuffield Department of Primary Care Health Sciences, University of Oxford, Oxford OX2 6GG, UK; brian.cook@phc.ox.ac.uk (B.C.); carmen.piernas-sanchez@phc.ox.ac.uk (C.P.); susan.jebb@phc.ox.ac.uk (S.A.J.)

**Keywords:** food choice, energy density, availability, labelling, socioeconomic position, online, supermarket

## Abstract

Reducing the energy density (ED) of product selections made during online supermarket food shopping has potential to decrease energy intake. Yet it is unclear which types of intervention are likely to be most effective and equitable. We recruited 899 UK adults of lower and higher socioeconomic position (SEP) who completed a shopping task in an online experimental supermarket. Participants were randomised in a 2 × 2 between-subjects design to test the effects of two interventions on the ED of shopping basket selections: labelling lower-ED products as healthier choices and increasing the relative availability of lower-ED products within a range (referred to as proportion). Labelling of lower-ED products resulted in a small but significant decrease (−4.2 kcal/100 g, 95% CIs −7.8 to −0.6) in the ED of the shopping basket. Increasing the proportion of lower-ED products significantly decreased the ED of the shopping basket (−17 kcal/100 g, 95% CIs −21 to −14). There was no evidence that the effect of either intervention was moderated by SEP. Thus, both types of intervention decreased the ED of foods selected in an online experimental supermarket. There was no evidence that the effectiveness of either intervention differed in people of lower vs. higher SEP.

## 1. Introduction

Lower dietary energy density (ED) is associated with lower bodyweight [1,2] and weight loss in people with obesity [3]. Reducing the ED of food products has been shown to decrease daily energy intake in experimental studies [4,5], suggesting that scalable interventions to reduce dietary ED could benefit population health. Recent reports suggest that the number of people ordering food from supermarkets online has grown rapidly [6,7]. A small number of studies testing interventions in online supermarkets have shown that offering shoppers healthier product “swaps” and positioning healthier food products more prominently can improve the nutritional quality of food purchases [8,9,10].

A common type of nutrition-based intervention is the use of information provision (e.g., labelling of lower-energy products) to alter choice behaviour [11]. A different approach is to alter the structural properties of the environment in which consumers make choices (e.g., increasing the proportion of products that are lower in energy) [12]. It has been proposed that because information provision approaches rely on conscious effort and motivation, they may be less equitable than structural-based interventions and therefore may widen socioeconomic position (SEP) inequalities in health [13,14,15]. For example, because there is evidence that lower-SEP groups are less motivated by health when making food choices [16], health-based information provision may have a significantly smaller effect on the diet of lower- compared to higher-SEP groups. However, there has been little direct testing of this proposition [14]. Two recent online experiments using virtual restaurant ordering paradigms found no overall evidence in support of this hypothesis as the effects that both an information provision intervention (energy labelling on menus) and a structural-based intervention (increasing the proportion of lower-energy menu options) had on total energy did not differ based on participant SEP [17,18].

The aim of the present study was to examine how an information provision intervention (labelling of lower-ED food products) and a structural intervention (increasing the proportion of lower-ED options within a range) affect the ED of shopping baskets among participants of lower vs. higher SEP in an online experimental supermarket. We hypothesised that increasing the proportion of lower-ED options would decrease the average ED of shopping basket selections and this effect would be similar in lower- and higher-SEP participants. We also hypothesised that any beneficial effect of labelling lower-ED food products may be dependent on SEP. We predicted that because lower SEP is associated with being less motivated by health when making food choices, labelling may have a smaller effect among lower-SEP participants.

## 2. Materials and Methods

### 2.1. Settings

This study was a randomised controlled online trial conducted in the Qualtrics survey platform and in an online experimental supermarket platform developed by the University of Oxford. The website is a research platform that recreates an online supermarket designed to support experiments into health behaviour and nudging. The site has all of the essential features of a normal online supermarket: browse and search for products, add products to the basket and check out, and has been used in previous research work [8,9,10]. It contains a grocery product range (FoodDB database [19]) including products names and images, department, aisle and shelf of the products, product size and nutritional information (energy, fat, saturated fat, carbohydrates, sugar, salt, fibre and protein). For the purpose of the present study, drinks were removed from the online supermarket as prior work has suggested it is inappropriate to combine food and drinks when calculating ED [20]. Products with missing size or energy were also excluded as these values were needed to calculate the average ED of shopping baskets. The final grocery product range included 10,434 food products. This trial was pre-registered at Clinicaltrials.gov (NCT04340791).

### 2.2. Participants

Participants were recruited through the platform Prolific Academic [21] in May 2020. Participants were eligible to participate if they were UK residents, aged 18 or above, fluent in English, had access to a computer with an internet connection, were the main or shared grocery shopper for their household and had no dietary restrictions. We intended to recruit a sample stratified by gender (approximately 50/50) and highest educational qualification (approximately 60% A level or below, 40% above A− level) to be broadly representative of the adult population in England [22]. Eligible participants who completed the study received monetary compensation in return for their participation. The study was approved by the Health and Life Sciences Research Ethics Committee at the University of Liverpool (reference: 4612) and the Medical Sciences Inter-Divisional Research Ethics Committee (IDREC) at the University of Oxford (Ref #R65010/RE001). Informed consent was obtained from all the participants. All participants were informed that the purpose of the study was to understand how different people made food purchases when shopping online.

### 2.3. Design

In a 2 × 2 between-subjects design, participants were randomly allocated to one of the following conditions: “standard proportion of lower-energy density products” and “no energy labelling” (P− L−), “standard proportion of lower-energy density products” and “energy labelling” (P− L+), “increased proportion of lower-energy density products” and “no energy labelling” (P+ L−) and “increased proportion of lower-energy density products” and “energy labelling” (P+ L+). Randomisation with 1:1:1:1 allocation was performed through the Qualtrics survey platform.

### 2.4. Online Shopping Task

Participants were asked to complete an online shopping task using a pre-determined shopping list of 10 items, as in previous studies using an online experimental supermarket [8,9,10]. When entering the website, participants were instructed to buy all the items on the list, selecting foods that they and their household would be likely to eat (see Supplementary File 1 for complete instructions). It was possible for a participant to select more or less than 10 items from the website but instructions explicitly mentioned that further items were not required in addition to the 10 items on the shopping list. The 10 foods were chosen from food groups that were major contributors to energy intake in the UK [23] and that included products with a range of ED. The list comprised of the following:

- A packet of biscuits;
- A standard-sized loaf of bread;
- A chilled pizza;
- An ice cream tub;
- A chilled ready meal for one;
- A pack of sausages;
- A sharing bag of crisps or savoury snacks;
- A pre-packed piece of cheese;
- A pack of yogurts;
- A jar of jam or sweet spread.

### 2.5. Interventions

The interventions were only applied in the sections of the website that included the food items from the shopping list. Lower-ED food products were defined in relation to a threshold for each food category from the shopping list: lower ED ≤ median of ED distribution within a food category.

**Figure 1:**
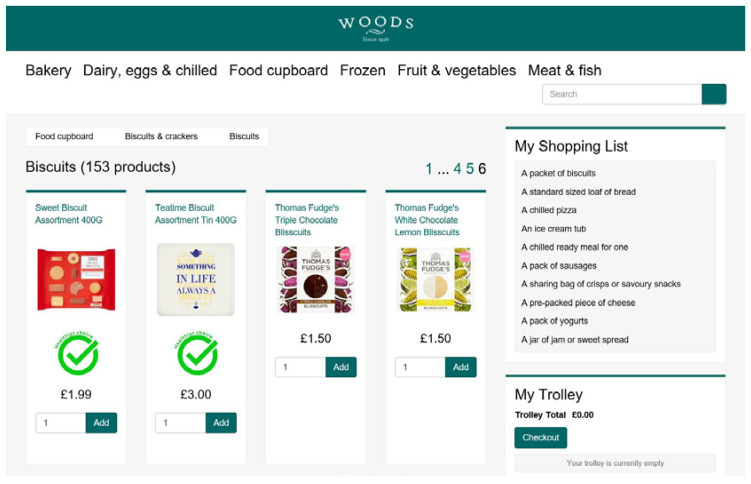
An example of a page of the online supermarket where “healthier choice” badges were displayed.

In the labelling conditions (L+), “healthier choice” badges in the form of a green tick were added next to the image of the lower-ED food products. In the labelling conditions only, instructions when entering the website explained the badges to the participants: “In the online supermarket, the green tick allows you to see which products (e.g., muesli) in each product category (e.g., cereals) are healthier choices with fewer calories per gram than most other products in the same category.” The design of the badge was based on previous research investigating the effect of front-of-package nutrition labelling on food choices [24]. Figure 1 shows a page of the online supermarket where badges were displayed.

In the increased proportion conditions (P+), the proportion of lower-ED vs. higher-ED options was reversed (67% lower ED and 33% higher ED) relative to the comparator conditions (P−) (33% lower ED and 67% higher ED). For each section of the website, the P− grocery set included the food products from the 1st, 5th, 7th, 8th, 9th and 10th ED deciles (9763 products) and the P+ grocery set included food products from the 1st, 2th, 3th, 4th, 6th and 10th ED deciles (9767 products). Products with the highest and the lowest ED (1st and 10th ED deciles) were the same across all conditions.

### 2.6. Outcomes

The primary outcome was the ED of the shopping basket calculated as the total energy purchased (kcal) divided by the total weight of the basket (g) and multiplied by 100. We also considered as secondary outcomes: total energy (kcal), energy from sugar (%), energy from saturated fat (SFA) (%), salt content (g/100 g), cost (GBP) and the percentage of lower-ED food items in the shopping basket.

### 2.7. Measures of SEP

We chose the primary measure of SEP to be education level because higher education level is associated with greater use of nutritional labels [25,26]. We collected two distinct measures of education level: highest educational qualification and total years in higher education. Highest educational qualification was measured using the question “What is your highest educational qualification?” See Supplementary File 2 for full response options. A level or below qualifications were categorised as “lower education level”, whereas qualifications above A level were categorised as “higher education level”. Years in higher education was measured using the question “After leaving school (i.e., at 16 years old), how many further years of higher education (i.e., a formal course) did you study for?” To account for both the level of qualification achieved and time spent in education, we calculated a continuous composite score (“level of education”) as the mean of the z-scores for highest educational level and years in higher education.

Participants were also asked to report the annual after-tax income of their household including all earners to the nearest GBP 1000. Equivalised household income [27] was calculated by dividing the after-tax household income by the sum of the equivalence value of all the household members (first adult = 1, additional adult or child aged 14 and over = 0.5, child aged 0–13 = 0.3). To measure perceived SEP, participants rated where they believed they stood in society from 1 (people who have the least money, least education and the worst jobs or no job) to 10 (people who have the most money, most education and the best jobs), using the MacArthur scale of subjective social status (SSS) [28].

### 2.8. Health and Weight Control Motives

To assess participants’ health and weight control motives in their food choices, we used the health (6 items—Cronbach’s α = 0.86) and weight control (4 items—Cronbach’s α = 0.83) subscales from the Food Choice Questionnaire developed by Steptoe et al., 1995 [29], with responses ranging from 1 = Not at all important to 4 = Very important.

### 2.9. Additional Measures

Age, gender, ethnic group, employment status, height, weight, dieting status and supermarket grocery shopping and online grocery shopping habits were recorded. Self-reported body mass index (BMI) was calculated in kg/m^2^. As participants were recruited during the COVID-19 pandemic lockdown period in the UK, participants were asked if they suspected having or having had COVID-19 and how worried they were about their health.

### 2.10. Procedure

Participants first answered a series of demographic questions including measures of SEP on Qualtrics. They were randomised in Qualtrics to one of the four experimental conditions and redirected to the online experimental supermarket website to perform the shopping task. On completion, participants were redirected back to Qualtrics where they completed the measures of health motives and a questionnaire about the validity of the online shopping task.

### 2.11. Statistical Analyses

We followed a pre-registered analysis protocol and the data presented in this study are openly available in Open Science Framework at DOI 10.17605/OSF.IO/KQ9C8, reference number KQ9C8. Only participants who completed the study were included in the analyses. Participants who failed at least one attention check (e.g., “How many times have you visited the planet Mars?”) were excluded. We analysed data from participants who bought at least 5 items out of 10 from the shopping list, and if participants bought more than the 10 items requested, as in previous research, we included all items bought [8,9,10].

The primary analysis was an ANCOVA testing the effect of labelling (categorical variable: L+ vs. L−), proportion (categorical variable: P+ vs. P−), level of education (continuous variable) and labelling*level of education and proportion*level of education interactions on the ED of the shopping basket. As exploratory analyses, we tested whether health and weight control motives moderated the effect of labelling and proportion on the total ED of the shopping basket. We calculated the Johnson–Neyman point defined as the value of a continuous moderator (health motives scores) for which the effect of an intervention (labelling) reaches statistical significance [30]. Sensitivity analyses were conducted to examine whether the pattern of results from the primary analysis differed: 1/after excluding participants guessing the aims of the study, 2/after excluding any participants who did not select 10 items in total and 1 item from each category on the shopping list, 3/substituting the composite score level of education by years in higher education as a continuous covariate, and by highest educational qualification split into lower vs. higher qualification levels. As secondary analyses, the primary analysis was replicated using two alternative measures of SEP (equivalised income and subjective social status). As secondary analyses, we also replicated the primary analysis on the ED of each food category from the shopping list and on the secondary outcomes. All statistical analyses described above were performed using SAS version 9.3 (SAS Institute, Inc., 2012 SAS^®^ 9.3. Cary, NC, USA). The Johnson–Neyman point was calculated using the PROCESS macro (Model 1) on SAS developed by Hayes [31]. Statistical tests level of significance was set at *p* < 0.05 for main and sensitivity analyses, and *p* < 0.01 in secondary analyses to account for multiple testing.

### 2.12. Sample Size

A previous study using the online supermarket platform showed a small-to-medium main effect (*f* = 0.14) of altering the default order and no significant main effect (*f* = 0.04) of offering swaps on the ED of the shopping basket [8]. We powered the study to detect small effects (*f* = 0.1) of the labelling and proportion interventions and of the interactions with level of education. In an ANCOVA including four groups (P− L−, P− L+, P+ L− and P+ L+) and one covariate (level of education), we required a sample size of 788 participants (197 per group) to detect a main effect of labelling or proportion or significant interaction effects with level of education, with 80% power at α = 0.05 (GPower 3.1).

## 3. Results

### 3.1. Participants

**Figure 2:**
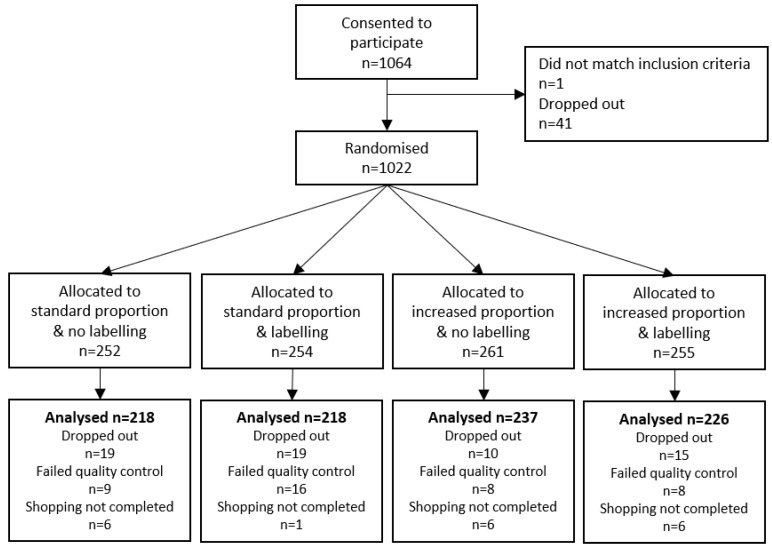
Flow chart.

A total of 1064 participants consented to participate. Data from 899 (84.5%) who completed the study were analysed (Figure 2). Participants’ characteristics are presented in Table 1. Participants bought on average 10.9 ± 4.8 items (with a 5% quantile of 9 and a 95% quantile of 15). A total of 720 participants bought exactly 10 items and, among them, 473 participants bought all the 10 items from the shopping list (based on the section of the online supermarket items were selected from). The number of participants who bought at least one item by shopping list category is shown in Supplementary File 3.

### 3.2. Effect of the Interventions and SEP on Online Grocery Shopping

On average across the four conditions, the ED of the shopping basket was 251 ± 29 kcal per 100 g. It was 249 ± 29 in the labelling conditions (L+, *n* = 444) and 253 ± 29 in the no labelling conditions (L−, *n* = 455), and it was 243 ± 26 in the increased proportion conditions (P+, *n* = 463) and 260 ± 30 in the standard proportion conditions (P−, *n* = 436).

We found a significant effect of labelling (F(1, 893) = 5.14, *p* = 0.024, partial η2 = 0.0057) and a significant effect of proportion (F(1, 893) = 86.39, *p* < 0.001, partial η2 = 0.0882), but no effect of level of education (F(1, 893) = 0.05, *p* = 0.815, partial η2 = 0.0001), nor of the interactions (labelling*level of education: F(1, 893) = 0.06, *p* = 0.806, partial η2 = 0.0001, proportion*level of education: F(1, 893) = 0.06, *p* = 0.814, partial η2 = 0.0001) on the ED of the shopping basket. As exploratory analyses, we included the interaction labelling*proportion in the previous model. The interaction was not significant (F(1, 892) = 0.05, *p* = 0.822, partial η2 = 0.0001) and the pattern of results for the other factors did not change. The effect of the interventions on the shopping basket across the four experimental conditions is reported in Table 2.

In secondary analyses, the effect of the interventions on the total energy content of the shopping basket was only significant for the proportion intervention. The proportion intervention increased the energy from sugar and decreased the energy from saturated fat of the shopping basket. The interventions did not significantly influence the total cost of the shopping basket. Replicating the main analysis on each food category from the shopping list, we found a significant effect of labelling on ED for only three of the 10 categories (ready meals, sausages and crisps) and a significant effect of proportion on ED for all categories but one (jam and spreads) (see Supplementary File 4).

Sensitivity analyses showed only a marginal effect of labelling on the ED of the shopping basket, but consistently with the primary analyses showed a significant effect of proportion and no effect of education or interactions (see Supplementary File 5). Moreover, the interaction effects remained non-significant when substituting level of education with equivalised income and subjective social status (see Supplementary File 6). In addition, the pattern of results remained the same when controlling for suspecting having coronavirus and for health concerns (see Supplementary File 7).

### 3.3. Effect of the Interventions and Health Motives on Online Grocery Shopping

We next explored whether either of the effects of the labelling and proportion interventions on the ED of the shopping basket were moderated by health and weight control motives (see Supplementary File 8). The mean score for health motives was 2.58 ± 0.65 (out of 4), and it was significantly correlated with level of education (*r* = 0.14, *p* < 0.001). The mean score for weight control motives was 2.21 ± 0.75 (out of 4), and it was not significantly correlated with level of education (*r* = −0.03, *p* = 0.340). We found a significant interaction between labelling and health motives. Using the Johnson–Neyman technique, we identified that the effect of the labelling intervention was significant only for participants with a health motives score above 2.74 at α = 0.01, corresponding to 42.2% of the sample. There was no significant interaction of health motives with proportion. In the model including weight control motives as a moderator, there was no significant interaction with proportion nor with labelling

### 3.4. Online Shopping Task Questionnaire

Among the participants allocated to the L+ conditions, 72% correctly identified that the “healthier choice” badge meant “Fewer calories per gram option” and no difference was found between participants from lower (72%) and higher education level (71%, chi-square test *p* = 0.846). Participants in L+ conditions reported that their shopping was influenced by how many calories they thought were in the options available to a larger extent than participants allocated to the L− conditions. The items from the shopping list were familiar to participants (see Supplementary File 9).

## 4. Discussion

The main aim of the present study was to examine the effect of labelling lower-ED products and increasing the relative availability (referred to as proportion) of lower-ED products on the ED of the shopping basket in an online supermarket shopping task. In primary analyses, both interventions resulted in a significant reduction in the ED of the shopping basket. The other main aim of the present study was to examine whether the effect that the two interventions had on the ED of the shopping basket was dependent on participant SEP. There was no evidence that the effect of either intervention differed among people from lower or higher SEP.

Increasing the proportion of lower-ED products had a sizeable effect on the ED and total energy of the shopping basket, most likely because the proportion of lower-ED products selected increased markedly from 39% to 62%. In the present study, the increased proportion of lower-ED products resulted in a decrease in energy from saturated fat but in an increase in energy from sugar. Labelling had a significant effect on the ED of the shopping basket but no overall effect on total energy selected, presumably because labelling was less effective in increasing the proportion of products selected that were lower in ED (48% to 52%). Importantly, both labelling lower-ED food products as healthier choices and increasing their proportion were associated with a reduced ED of the shopping basket, suggesting that both types of interventions have some benefit. Although both nutritional labelling and increasing the availability of healthier food have been shown to promote healthier food choices in the out-of-home food sector [11,12], evidence regarding their effects on food purchasing behaviour online is scarce. This is the first study to our knowledge to investigate the effects of both types of interventions on online grocery shopping.

It has been hypothesised that information provision-based interventions (e.g., labelling of lower-ED products) may widen inequalities in health by being less effective in lower-SEP groups [14,15], but in the present study, there was no evidence in support of this. Likewise, the effect that increasing the proportion of lower-ED products had on the ED of the shopping basket was not dependent on SEP. It has been suggested that information provision-based interventions may reinforce socioeconomic inequalities because they require individuals to use their cognitive, psychological and material resources, which tend to be socioeconomically patterned [13]. In our experiment, the green tick label appeared to be well understood by the participants and no differences were found across SEP for understanding the label. Moreover, the total cost of the shopping basket did not differ in the labelling compared to the no labelling condition, even though the basket ED was reduced. These results suggest that when labelling is prominent, easy to understand and when there is no cost barrier in choosing healthier options, food labelling may be an equitable intervention for higher- and lower-SEP populations. However, in exploratory analyses, we found that the effect of labelling on the ED of the shopping basket was observed only among participants who reported being motivated by health in their day-to-day food choices. Conversely, there was no evidence that the effect of increasing availability on the ED of the shopping basket was dependent on food choice motives. These findings suggest increasing the relative availability of lower-ED products is likely to be a more far reaching approach to increasing the nutritional quality of food purchases, whereas labelling of lower-ED products was primarily observed among those who are motivated by health.

There are limitations to the present research. Although the online supermarket shopping environment has been used in previous research [8,9,10], participants made hypothetical product selections and did not spend money. There is some evidence that hypothetical purchasing tasks administered virtually relate to purchasing behaviour in the real world [34,35], but replicating the present findings in real-world settings would be informative. Furthermore, we used a selective labelling approach with only some products (based on ED) receiving the green tick as opposed to labels depicting healthiness for all products. The labelling was prominent in the online supermarket and effects may be smaller if real-world implementation resulted in labelling being less prominent. We only examined choice behaviour for food products and not drinks, although we are not aware of any reason why findings would be expected to differ. The sample size collected meant we were powered to detect a relatively small moderating effect of intervention effectiveness by SEP, but there was not significant moderation in analyses. We did find evidence that being motivated by health in dietary choices moderated the effectiveness of labelling, but even among participants scoring higher in health-based food choice motives, the effect of labelling was still relatively small. Lower SEP was significantly associated with reporting being less motivated by health in day-to-day dietary choices, but this association was small (*r* = 0.14). Given the size of these associations, if there is an SEP–labelling intervention effectiveness moderation effect driven by SEP differences in food choice motives, it would presumably be very small in size. Finally, we characterised SEP based on participant highest educational qualification in main analyses and SEP can be considered a multi-facetted construct. However, results were the same when examining other markers of SEP (i.e., household income, subjective social status).

## 5. Conclusions

Labelling lower-ED products as healthier choices and increasing the proportion of lower-ED products both decreased the ED of foods selected in an online experimental supermarket. There was no evidence that the effectiveness of either intervention differed in people of lower vs. higher SEP.

## Figures and Tables

**Table 1:** Participants characteristics.

Variables	All Participants (*n* = 899)
Age, Years, Mean (SD)	39.5 (13.1)
Gender, female, *n* (%)	460 (51.2)
Ethnicity, white, *n* (%)	837 (93.1)
Employment status, *n* (%)	
Full or part-time	669 (74.4)
Student	15 (1.7)
Retired	70 (7.8)
Other unemployed	145 (16.1)
Highest educational qualification, *n* (%)	
No formal qualification	23 (2.5)
1–3 GCSEs	77 (8.6)
4+ GCSEs	177 (19.7)
A level	283 (31.5)
Certificate of higher education (CertHE)	33 (3.7)
Diploma of higher education (DipHE)	41 (4.5)
Bachelor’s degree	183 (20.4)
Master’s degree	65 (7.2)
Doctorate	17 (1.9)
Years of higher education, mean (SD)	2.8 (2.5)
Equivalised income, GBP, mean (SD)	22,139 (18,549)
Subjective social status, mean (SD)	5.2 (1.6)
BMI, kg/m^2^, mean (SD)	27.0 (5.8)
Missing or implausible ^1^, *n* (%)	9 (1)
Dieting status, yes, *n* (%)	90 (10)
Supermarket shopping/week, GBP, mean (SD)	78.9 (44.3)
Supermarket grocery shopping frequency, *n* (%)	
Less than once a month	37 (4.1)
1–3 times per month	162 (18.0)
1–2 times per week	558 (62.1)
3–4 times per week	114 (12.7)
5 times per week or more often	28 (3.1)
Online grocery shopping frequency, *n* (%)	
Never or not in the last year	240 (26.7)
1–3 times in the last year	246 (27.4)
4–11 times in the last year	227 (25.3)
1–3 times per month	118 (13.1)
Once per week or more often	68 (7.6)

^1^ Excluding weight <30 kg or >250 kg, height <1.45 m or >3 m [32,33].

**Table 2:** Energy density and nutrient content of the shopping baskets across the four experimental conditions.

	P−/L− (*n* = 218)	P−/L+ (*n* = 218)	P+/L− (*n* = 237)	P+/L+ (*n* = 226)	Labelling L+ vs. L−	Proportion P+ vs. P−
ED (kcal/100 g)	262 (29)	257 (30)	245 (27)	241 (24)	−4.2 (−7.8 to −0.6)	−17 (−21 to −14)
Total energy (kcal)	11295 (4274)	10753 (3943)	9980 (2005)	10397 (4802)	−67 (−734 to 601)	−852 (−1519 to −184)
Energy from sugar (%)	4.83 (1.21)	5.02 (1.18)	5.42 (1.26)	5.57 (1.38)	0.17 (−0.04 to 0.39)	0.57 (0.36 to 0.79)
Energy from SFA (%)	5.48 (1.07)	5.44 (1.26)	4.91 (1.06)	4.80 (1.04)	−0.07 (−0.26 to 0.12)	−0.60 (−0.79 to −0.41)
Salt content (g/100 g)	0.74 (0.11)	0.74 (0.10)	0.74 (0.09)	0.76 (0.15)	0.01 (−0.01 to 0.03)	0.01 (−0.01 to 0.03)
Number of products	11.4 (6.61)	11.0 (4.94)	10.3 (1.60)	11.0 (4.89)	0.21 (−0.62 to 1.04)	−0.58 (−1.41 to 0.26)
Lower-ED choices (%)	35.7 (15.4)	42.3 (18.5)	60.1 (17.2)	63.1 (17.3)	4.77 (1.81 to 7.73)	22.6 (19.7 to 25.6)
Cost (GBP)						
Total	20.7 (9.35)	20.6 (7.69)	18.6 (3.73)	19.8 (6.23)	0.59 (−0.62 to 1.80)	−1.42 (−2.63 to −0.21)
/100 g	0.48 (0.11)	0.50 (0.11)	0.47 (0.10)	0.48 (0.10)	0.02 (−0.002 to 0.03)	−0.02 (−0.04 to −0.003)
/100 kcal	0.19 (0.05)	0.20 (0.04)	0.19 (0.05)	0.20 (0.05)	0.01 (0.001 to 0.02)	0.005 (−0.003 to 0.01)

Values are means (SDs) in the first four columns and parameters estimates (95% CI for energy density (ED) and 99% CIs for secondary outcomes; CIs not crossing zero indicate significant difference) for labelling and proportion factors from the models including labelling, proportion, level of education, level of education*labelling and level of education*proportion as predictors in the next two columns. SFA = saturated fat.

## Supplementary Materials

The following are available online at https://www.mdpi.com/2072-6643/12/12/3618/s1, supplementary file 1. Online shopping task instructions, supplementary file 2. Highest educational qualification measure, supplementary file 3. Shopping for individual items from the shopping list, supplementary file 4. ED by food categories across experimental conditions, supplementary file 5. Sensitivity analyses, supplementary file 6. Secondary analyses with alternatives measures of SEP, supplementary file 7. COVID-19-related analyses, supplementary file 8. Effect of the interventions and health motives on online grocery shopping, supplementary file 9. Analyses on the online shopping task questionnaire.
