# Supplementary material for: Effects of Labelling and Increasing the Proportion of Lower-Energy Density Products on Online Food Shopping: A Randomised Control Trial in High- and Low-Socioeconomic Position Participants
Source: Nutrients. 2020 Nov 25;12(12):3618. doi: 10.3390/nu12123618 (PMC7760499; doi:10.3390/nu12123618)
Supplement: Supplementary file 1 [file nutrients-12-03618-s001.zip › supplementary new/supplementary file 1 new.docx]

1. **Online shopping task instructions**

When entering the online supermarket participants were shown the following text: “We would like you to do online grocery shopping on a supermarket website. This is not a real commercial site, and you will not be asked to spend your own money. You are given a shopping list and we ask you to buy all the items on the list. You can cross out the items on the list once you have added them to your trolley. You do not need to buy additional items. To search for the items from the shopping list, you can either go through the website categories or use the searching bar by copy-pasting the name of the shopping list item. When doing the shopping task, please select foods you and your household would be likely to choose.”
