# Supplementary material for: Effects of Labelling and Increasing the Proportion of Lower-Energy Density Products on Online Food Shopping: A Randomised Control Trial in High- and Low-Socioeconomic Position Participants
Source: Nutrients. 2020 Nov 25;12(12):3618. doi: 10.3390/nu12123618 (PMC7760499; doi:10.3390/nu12123618)
Supplement: Supplementary file 1 [file nutrients-12-03618-s001.zip › supplementary new/supplementary file 2 new.docx]

**2 Highest educational qualification measure**

Highest educational qualification was measured using the question “What is your highest educational qualification? If you are a student, please select the qualification you are currently studying for.” Participants selected one of the following options, coded from 1 to 9: No formal qualifications, 1-3 GCSEs or equivalent, 4+ GCSEs or equivalent, A level or equivalent, Certificate of higher education (CertHE) or equivalent, Diploma of higher education (DipHE) or equivalent, Bachelor’s degree or equivalent, Master’s degree or equivalent, Doctoral degree or equivalent.
