# Supplementary material for: Effects of Labelling and Increasing the Proportion of Lower-Energy Density Products on Online Food Shopping: A Randomised Control Trial in High- and Low-Socioeconomic Position Participants
Source: Nutrients. 2020 Nov 25;12(12):3618. doi: 10.3390/nu12123618 (PMC7760499; doi:10.3390/nu12123618)
Supplement: Supplementary file 1 [file nutrients-12-03618-s001.zip › supplementary new/supplementary file 3 new.docx]

**3. Shopping for individual items from the shopping list**

During the online shopping task, all the participants did not buy all the items from the shopping list. **Table S1** shows the number of participants who bought each item.

**Table S1.** Number of participants who bought each item from the shopping list

| **Shopping list items** | **Participants who bought the items (n)** |
| --- | --- |
| A packet of biscuits | 840 |
| A standard sized loaf of bread | 860 |
| A chilled pizza | 797 |
| An ice cream tub | 854 |
| A chilled ready meal for one | 774 |
| A pack of sausages | 805 |
| A sharing bag of crisps or savoury snacks | 805 |
| A pre-packed piece of cheese | 880 |
| A pack of yogurts | 838 |
| A jar of jam or sweet spread | 843 |
