# Supplementary material for: Effects of Labelling and Increasing the Proportion of Lower-Energy Density Products on Online Food Shopping: A Randomised Control Trial in High- and Low-Socioeconomic Position Participants
Source: Nutrients. 2020 Nov 25;12(12):3618. doi: 10.3390/nu12123618 (PMC7760499; doi:10.3390/nu12123618)
Supplement: Supplementary file 1 [file nutrients-12-03618-s001.zip › supplementary new/supplementary file 4 new.docx]

**4. ED by food categories across experimental conditions**

We replicated the primary analysis individually on ED of the food items bought from each food category of the shopping list (**Table S2**).

**Table S2.** ED (kcal/100g) of the food items purchased from each category of the shopping list across the four experimental conditions

|  | **P-/L-** | **P-/L+** | **P+/L-** | **P+/L+** | **L+ vs. L-** | **P+ vs. P-** |
| --- | --- | --- | --- | --- | --- | --- |
| Biscuits | 492  (30) | 486  (38) | 464  (43) | 462  (46) | -3.7  (-11 to 3.4) | -26  (-33 to -19) |
| Bread | 257  (23) | 253  (23) | 244  (24) | 243  (22) | -2.1  (-6.2 to 1.9) | -11  (-15 to -7.0) |
| Pizza | 272  (27) | 269  (26) | 257  (25) | 252  (23) | -4.4  (-9.0 to 0.2) | -16  (-21 to -11) |
| Ice cream | 233  (53) | 239  (50) | 229  (63) | 225  (61) | 1.8  (-8.3 to 12) | -9.0  (-19 to 1.2) |
| Ready meal | 142  (31) | 132  (31) | 109  (21) | 107  (14) | -6.2  (-11 to -1.6) | -29  (-34 to -25) |
| Sausages | 300  (56) | 290  (63) | 258  (66) | 239  (61) | -14  (-26 to -3.3) | -46  (-57 to -35) |
| Crisps | 508  (29) | 502  (34) | 491  (38) | 484  (36) | -6.5  (-13 to -0.2) | -17  (-23 to -11) |
| Cheese | 389  (41) | 371  (48) | 360  (76) | 361  (78) | -8.3  (-19 to 2.8) | -20  (-31 to -8.6) |
| Yogurts | 101  (26) | 98  (27) | 80  (35) | 75  (35) | -4.0  (-9.5 to 1.5) | -21  (-27 to -16) |
| Jam | 264  (114) | 270  (125) | 262  (56) | 266  (59) | 5.7  (-11 to 22) | -2.9  (-20 to 14) |

Values are means (SDs) in the first four columns and parameters estimates (99% Cis. CIs not crossing zero indicate significant difference) for labelling and proportion factors from the models including labelling, proportion, level of education, level of education*labelling, level of education*proportion as predictors in the next two columns.
