# Supplementary material for: Effects of Labelling and Increasing the Proportion of Lower-Energy Density Products on Online Food Shopping: A Randomised Control Trial in High- and Low-Socioeconomic Position Participants
Source: Nutrients. 2020 Nov 25;12(12):3618. doi: 10.3390/nu12123618 (PMC7760499; doi:10.3390/nu12123618)
Supplement: Supplementary file 1 [file nutrients-12-03618-s001.zip › supplementary new/supplementary file 5 new.docx]

**5. Sensitivity analyses**

Sensitivity analyses were conducted replicating the primary analysis ANCOVA model after excluding 121 participants having guessed the aims of the study (Model 1), excluding participants who did not select 10 items in total and 1 item from each category of the shopping list (Model 2), and substituting the composite score level of education by years in higher education as a continuous covariate (Model 3) and by highest educational qualification split into lower vs. higher qualification levels (Model 4 – 2x2x2 ANOVA) **(Table S3)**. The four sensitivity analyses models showed only a marginal effect of labelling on ED of the shopping basket, but consistently with the primary analysis, they showed a significant effect proportion and no effect of education nor of the interactions.

**Table S3.** Description of the sensitivity analyses models, dependent variable ED of the shopping basket

|  | ***F*** | ***p*** | ***partial η^2^*** |
| --- | --- | --- | --- |
| **Model 1 (n=778)**  Labelling  Proportion  Level of education  Level of education*labelling  Level of education*proportion | 2.87  65.02  0.09  1.14  0.01 | 0.091  < 0.001  0.765  0.286  0.929 | 0.0037  0.0777  0.0001  0.0015  < 0.0001 |
| **Model 2 (n=473)**  Labelling  Proportion  Level of education  Level of education*labelling  Level of education*proportion | 2.64  144.46  0.64  < 0.01  < 0.01 | 0.105  < 0.001  0.423  0.950  0.948 | 0.0056  0.2363  0.0014  < 0.0001  < 0.0001 |
| **Model 3 (n=899)**  Labelling  Proportion  Years in higher education  Years in higher education*labelling  Years in higher education*proportion | 2.53  37.11  0.39  0.01  0.01 | 0.112  < 0.001  0.535  0.905  0.940 | 0.0028  0.0399  0.0004  < 0.0001  < 0.0001 |
| **Model 4 (n=899)**  Labelling  Proportion  Highest educational qualification  Highest educational qualification*labelling  Highest educational qualification*proportion | 3.31  81.95  0.01  2.38  < 0.01 | 0.069  < 0.001  0.923  0.123  0.960 | 0.0037  0.0841  < 0.0001  0.0027  < 0.0001 |
