# Supplementary material for: Effects of Labelling and Increasing the Proportion of Lower-Energy Density Products on Online Food Shopping: A Randomised Control Trial in High- and Low-Socioeconomic Position Participants
Source: Nutrients. 2020 Nov 25;12(12):3618. doi: 10.3390/nu12123618 (PMC7760499; doi:10.3390/nu12123618)
Supplement: Supplementary file 1 [file nutrients-12-03618-s001.zip › supplementary new/supplementary file 6 new.docx]

**6. Secondary analyses with alternatives measures of SEP**

The primary analysis was replicated using two alternative measures of socioeconomic position (equivalised income and subjective social status) and no interaction effect was found between these measures and the interventions (labelling and proportion) (**Table S4**).

**Table S4.** Description of the models, dependent variable: ED of the shopping basket

|  | ***F*** | ***p*** | ***partial η^2^*** |
| --- | --- | --- | --- |
| **Equivalised income (n=897^a^)**  Labelling  Proportion  Income  Income*labelling  Income*proportion | 1.55  37.89  0.45  0.01  2.21 | 0.213  < 0.001  0.504  0.920  0.137 | 0.0017  0.0408  0.0005  < 0.0001  0.0025 |
| **Subjective Social Status (n=899)**  Labelling  Proportion  SSS  SSS*labelling  SSS*proportion | 0.39  14.66  0.40  1.76  1.49 | 0.531  < 0.001  0.525  0.186  0.223 | 0.0004  0.0162  0.0005  0.0020  0.0017 |

^a^ Excluding two outliers: 388888.9 and 144444.4, all the other values <88100
