# Supplementary material for: Effects of Labelling and Increasing the Proportion of Lower-Energy Density Products on Online Food Shopping: A Randomised Control Trial in High- and Low-Socioeconomic Position Participants
Source: Nutrients. 2020 Nov 25;12(12):3618. doi: 10.3390/nu12123618 (PMC7760499; doi:10.3390/nu12123618)
Supplement: Supplementary file 1 [file nutrients-12-03618-s001.zip › supplementary new/supplementary file 7 new.docx]

**7. COVID-19-related analyses**

Six participants declared that they suspected having COVID-19 when they completed the study and 97 participants declared that they suspected having had COVID-19 before. A total of 490 participants (54.5%) declared being slightly or very worried about their health on a scale from 1 to 4: 1 = not at all worried, 2 = not worried, 3 = slightly worried, 4 = very worried. The pattern of results remained the same when controlling for suspecting having coronavirus (categorical variable: yes or no) and for health concerns (continuous variable: 1 to 4); suspecting having coronavirus and health concern did not influence ED of the shopping basket (**Table S5**).

**Table S5.** Description of the model including COVID-19 suspicion and health concern as covariates, dependent variable: ED of the shopping basket

|  | ***F*** | ***p*** | ***partial η^2^*** |
| --- | --- | --- | --- |
| Labelling  Proportion  Level of education  Level of education*labelling  Level of education*proportion  COVID-19  Health concern | 5.04  86.41  0.03  0.06  0.06  0.19  0.05 | 0.025  <0.001  0.868  0.808  0.812  0.666  0.815 | 0.0056  0.0884  < 0.0001  0.0001  0.0001  0.0002  0.0001 |
