# Supplementary material for: Effects of Labelling and Increasing the Proportion of Lower-Energy Density Products on Online Food Shopping: A Randomised Control Trial in High- and Low-Socioeconomic Position Participants
Source: Nutrients. 2020 Nov 25;12(12):3618. doi: 10.3390/nu12123618 (PMC7760499; doi:10.3390/nu12123618)
Supplement: Supplementary file 1 [file nutrients-12-03618-s001.zip › supplementary new/supplementary file 8 new.docx]

**8. Effect of the interventions and health motives on online grocery shopping**

We explored whether health motives and weight control motives could individually moderate the effect of the interventions on ED of the shopping basket substituting level of education in the primary analysis ANCOVA model by health or weight control motives scores (**Table S6**).

**Table S6.** Description of the models, dependent variable ED of the shopping basket (n = 899)

|  | ***F*** | ***p*** | ***partial η^2^*** |
| --- | --- | --- | --- |
| **Healthiness motives**  Labelling  Proportion  Healthiness  Healthiness*labelling  Healthiness*proportion | 4.39  7.34  10.77  7.40  0.17 | 0.036  0.007  0.001  0.007  0.684 | 0.0049  0.0082  0.0119  0.0082  0.0002 |
| **Weight control motives**  Labelling  Proportion  Weight control  Weight control*labelling  Weight control*proportion | 0.12  17.77  23.60  0.09  1.57 | 0.731  <0.001  <0.001  0.765  0.211 | 0.0001  0.0195  0.0258  0.0001  0.0018 |
