# Supplementary material for: Effects of Labelling and Increasing the Proportion of Lower-Energy Density Products on Online Food Shopping: A Randomised Control Trial in High- and Low-Socioeconomic Position Participants
Source: Nutrients. 2020 Nov 25;12(12):3618. doi: 10.3390/nu12123618 (PMC7760499; doi:10.3390/nu12123618)
Supplement: Supplementary file 1 [file nutrients-12-03618-s001.zip › supplementary new/supplementary file 9 new.docx]

**9. Analyses on the online shopping task questionnaire**

At the end of the online experiment, participants were asked questions to assess the validity of the online shopping task (meaning of the badges in L+ conditions, energy content influence on grocery shopping, grocery shopping habits).

Among the participants allocated to the L+ conditions, 72.0 % declared that the “healthier choice” badge meant “Fewer calories per gram option”. No difference was found between participants from lower (72.3 %) and higher education level (71.4 %, chi-square test *p* = 0.846).

The participants were also asked to report whether their shopping was influenced by how many calories they thought were in the options available on a scale from 1 (strongly disagree) to 7 strongly agree. The ‘kcal influence’ scores in each experimental condition are: P-/L-: 2.39 ± 1.41, P-/L+: 2.90 ± 1.50, P+/L-: 2.57 ± 1.55, and P+/L+: 2.81 ± 1.39. We found a significant effect of labelling on the ‘kcal influence’ score (*F*(1, 893) = 14.70, *p* < 0.001, partial η^2^ = 0.0162), but no effect of proportion (*F*(1, 893) = 0.18, *p* = 0.667, partial η^2^ = 0.0002), no effect of education level (*F*(1, 893) = 0.04, *p* = 0.834, partial η^2^ < 0.0001) and no effect of the interactions (level of education*labelling: *F*(1, 893) = 0.84, *p* = 0.359, partial η^2^ = 0.0009, and level of education*proportion: *F*(1, 893) = 0.09, *p* = 0.760, partial η^2^ = 0.0001).

The participants were then asked whether they would normally buy each item from the shopping list on a scale from 1 (never) to 6 (very frequently) (**Table S7**). We calculated a global score for each participant as the mean across the 10 items. We found no effect of labelling on the score (*F*(1, 893) = 0.11, *p* = 0.743, partial η^2^ = 0.0001), no effect of proportion (*F*(1, 893) = 0.34, *p* = 0.558, partial η^2^ = 0.0004), but a significant effect of education level (*F*(1, 893) = 9.26, *p* = 0.002, partial η^2^ = 0.0103) and no effect of the interactions (level of education*labelling: *F*(1, 893) = 0.08, *p* = 0.779, partial η^2^ = 0.0001, and level of education*proportion: *F*(1, 893) < 0.01, *p* = 0.951, partial η^2^ < 0.0001). The items from the shopping list were less frequently bought by the participants of higher education level.

**Table S7.** Frequency of purchase for each item from the shopping list

|  | **Never** | **Very rarely** | **Rarely** | **Occasionally** | **Frequently** | **Very frequently** |
| --- | --- | --- | --- | --- | --- | --- |
| Biscuits | 17 (1.9) | 107 (11.9) | 94 (10.5) | 269 (29.9) | 267 (29.7) | 145 (16.1) |
| Bread | 9 (1.0) | 28 (3.1) | 27 (3.0) | 89 (9.9) | 315 (35.0) | 431 (48.0) |
| Pizza | 56 (6.2) | 105 (11.7) | 118 (13.1) | 301 (33.5) | 205 (22.8) | 114 (12.7) |
| Ice cream | 38 (4.2) | 201 (22.4) | 210 (23.4) | 314 (34.9) | 93 (10.3) | 43 (4.8) |
| Ready meal | 212 (23.6) | 210 (23.4) | 155 (17.2) | 171 (19.0) | 102 (11.4) | 49 (5.4) |
| Sausages | 43 (4.8) | 59 (6.6) | 82 (9.1) | 272 (30.2) | 318 (35.4) | 125 (13.9) |
| Crisps | 28 (3.1) | 84 (9.3) | 111 (12.4) | 273 (30.4) | 274 (30.5) | 129 (14.3) |
| Cheese | 18 (2.0) | 28 (3.1) | 32 (3.6) | 102 (11.3) | 354 (39.4) | 365 (40.6) |
| Yogurts | 44 (4.9) | 71 (7.9) | 56 (6.2) | 189 (21.0) | 265 (29.5) | 274 (30.5) |
| Jam & spreads | 48 (5.4) | 137 (15.2) | 175 (19.5) | 315 (35.0) | 169 (18.8) | 55 (6.1) |

Values are n (%)
